# Clinical Outcome of High Risk Gastrointestinal Stromal Tumor in a Meckel’s Diverticulum

**Published:** 2009-03

**Authors:** Michele Scialpi, Christian Franzini, Antonio Cavaliere, Francesco Barberini, Irene Piscioli, Ilaria Franceschetti, Luciano Lupattelli

**Affiliations:** 1 *Department of Surgery Radiology, Odontostomatology Science, Section of Diagnostic and Interventional Radiology, University of Perugia, S. Maria della Misericordia Hospital, S. Andrea delle Fratte, 06156 Perugia, Italy;*; 2 *Division of Surgery, Guastalla Hospital, Via Donatori di Sangue 1, 40016 Guastalla (RE), Italy;*; 3 *Department of Anatomic Pathology, University of Perugia, Via Brunamonti 51, 06100 Perugia, Italy;*; 4 *Oncological Surgical Unit, University of Perugia, Via Brunamonti 51, 06100 Perugia, Italy;*; 5 *Department of Radiology, Budrio Hospital, Via Benni 44, 44054 Budrio (BO), Italy;*; 6 *Institute of Anatomic Pathology, S. Maria del Carmine Hospital, Piazzale S.Maria 6, 38068 Rovereto (TN), Italy*

**Keywords:** gastrointestinal stromal tumor, Meckel diverticulum, imatinib mesylate

## Abstract

We describe a seven years follow-up of a high risk gastrointestinal stromal tumor in a Meckel’s diverticulum in a 68-year-old man with abdominal pain and vomiting. The patient was operated in emergency for peritonitis due to perforation of small intestine and treated with imatinib mesylate. The metastatic progression of the disease demonstrated the value of prognostic indicators (mitotic rate >10/50 high power field, necrosis and 8 cm in maximum diameter) for assessing risk of aggressive behaviour. Computed tomography was a valuable procedure for detection of local recurrence, the distant metastases and for surveillance after surgery in the follow-up. The review of the literature shows that this case has the longest follow up and consents the comparisons of the same neoplasm in other sites most frequent and better described than Meckel’s diverticulum.

## INTRODUCTION

Gastrointestinal stromal tumors (GISTs) may be defined as mesenchymal tumors that express KIT protein or have an activating mutation in a class III receptor tyrosine kinase gene, the PDGFR-α gene, which encodes the platelet derived growth factor receptor-alpha, a tyrosine kinase protein. The KIT protein can be detected by immunohistochemical assays for the CD117 antigen (1).

Incidence of GISTs within the Meckel’s diverticulum (MD) is 0.5 to 3.2% (2, 3). To our knowledge, only six cases have been previously reported. Presenting symptoms, radiologic and clinical findings have been described, but not the behaviour of the disease (Table 1) (2-12).

**Table 1:** Gastrointestinal stromal tumor in Meckel’s diverticulum: review of the literature

Authors	Year/Sex Presentation symptoms	Preoperative Radiology and Clinical Diagnosis	Surgical Specimen and Histological Diagnosis	Follow up

Johnston *et al* 2001 (2)	58/male.	**Abdominal and pelvic US:** solid, hypoechoic, rounded mass lying anterior to the bifurcation of the aorta and indenting the dome of the bladder. The mass had an echobright posterior wall consistent with calcification. **CT of the pelvis:** pelvic mass adjacent to loops of bowel in the mesentery, with close relation to the bladder. **PD:** complicated mesenteric cyst or a soft tissue sarcoma arising in the mesentery.	10 cm of small bowel containing the Meckel’s mass.	Not reported.
	20 hours history of right iliac fossa pain.		GIST not otherwise specified.	
Stolk *et al* 2002 (3),	50/male.	**Selective angiogram of branch of superior mesenteric artery**: pathologic vascular configuration just cranial to urinary bladder. **PD:** MD or tumor.	MD with 5 cm ulcer-like mass.	Not reported.
Biemans & Vos 2005 (4)	Melena for 5 days, dispnea on exertion, nausea.		Stromal tumor with central necrosis (5 mitoses per 2 square millimeters; CD 117 and CD 34 positivity).	
Lorusso *et al* 2003 (5)	55/male.	**Echo-color Doppler:** femoro-iliac-caval-sovrahepatic venous thrombosis.	3,5 in the MD.	Not reported.
	Edema to the legs.	**CT scan:** 12 cm mass of small intestine and MD. **Abdominal X-Ray:** MD with stenosis and dilatation of the small intestine.	GIST (positivity for CD117).	
Hager *et al* 2005 (6)	75/male.	**Abdominal X-Ray:** pneumoperitoneum	Excision of perforated MD with incomplete tumor resection.	Discharged 4 weeks after laparotomy because of post operative complications.
	Not reported.	**PD:** perforation of a hollow.	2 cm-spindle cell GIST, 1 mitosis per 50 high-power fields, Ki 67 2%.	
Mijandrusic Sincic *et al* 2005 (7)	81/male.	**Abdominal X-Ray:** dilated loops of intestine with large packers of gas.	5 cm segment of small bowel with a polypous tumor measuring 3 cm in diameter in a MD, 18 cm ileum, 20 cm cecum and ascending colon. Low risk GIST with Crohn’s disease in ileum and colon.	Discharged 13 days after admission.
	12 hours before the admission sudden colic pain, constipation, vomiting of fecal matter.	**Abdominal ultrasound:** distended bowel loops with anti-peristalsis. **PD:** iliac perforation.		
Khoury II *et al* 2006 (8)	28/male.	**CT with intravenous and oral contrast:** a small bowel obstruction with a complex mass contiguous to the obstruction, which was cystic with enhancing soft tissue components. **PD:** MD.	11.5 × 11 × 6.5 circumscribed mass arising from the MD wall.	Treatment with imatinib mesylate (outcome not specified).
	Severe abdominal pain of increasing intensity, nausea, emesis.		High risk GIST.	
Chandramohan *et al* 2007 (9)	65/male.	**Abdominal ultrasound:** 6 × 9 cm exophytic hypoechoic lesion in the pelvis near sigmoid colon.	MD tumor with 3 cm of ileum, involving the anterior wall of sigmoid colon and part of the urinary bladder musculature.	Uneventful postoperative period.
	Constipation for 4 months and bleeding per rectum for one month.	**Contrast enhanced CT scan:** lobulated mass in pelvis compressing anterior wall of sigmoid colon and located posterosuperior to the urinary bladder. **PD:** small bowel tumor compressing sigmoid colon.	GIST (2-3 mitosis/50 HPF, positivity for vimentin and CD 117).	
Komen *et al* 2007 (10)	79/male.	**Contrast enhanced CT scan:** large, well-circumscribed mass in the left upper abdomen.	14 cm mass arising from MD.	Not reported.
	Rectal bleeding.		GIST (CD117 positivity).	
Macaigne *et al* 2007 (11)	66/female.	**First surgery (1996):** not reported. **Second surgery (2004) CT scan:** 12×10×7,5 cm, abdominal, polilobulated, heterogeneous mass between the intestine, extended to the subcutaneous tissue. Bone, liver, axillary and abdominal nodal metastases.	First surgery: 3 cm MD tumor	Second surgery: surgical biopsy of the metastases (26/10 HPF, diffuse positivity for CD117, 50% positivity for CD34). Treatment with imatinib mesylate 2006: liver, nodal and peritoneal metastases.
	Rectal bleeding.		Ulcerated leiomyoma(1 mitose/50 HPF, CD34+) with retrospective diagnosis of GIST (positivity for CD117).	
De la Morena *et al* 2007 (12)	47/female	**Transvaginal ultrasound:** 6,2 × 4,8 cm mass likely of ovarian origin.	MD tumor.	Not evidence of disease five years after surgery.
	Severe abdominal pain, emesis.		GIST borderline (1 mitose/10 HPF, CD117 positive).	

CT, computed tomography; HPF, high power field; MD, Meckel’s diverticulum; PD, preoperative diagnosis; US, ultrasound.

We present the clinical outcome of a high risk GIST in a MD, treated with imatinib mesylate, with a follow-up of seven years, as a first example of “disease biologic progression model” suggested by prognostic indicators.

## CASE REPORT

A 68-year-old man was referred to our institution in August 2001 because of abdominal pain and vomiting.

A plain X-ray of the abdomen showed distension of the small intestine and colon; no air-fluid levels or subphrenic free air was revealed.

**Figure 1:**
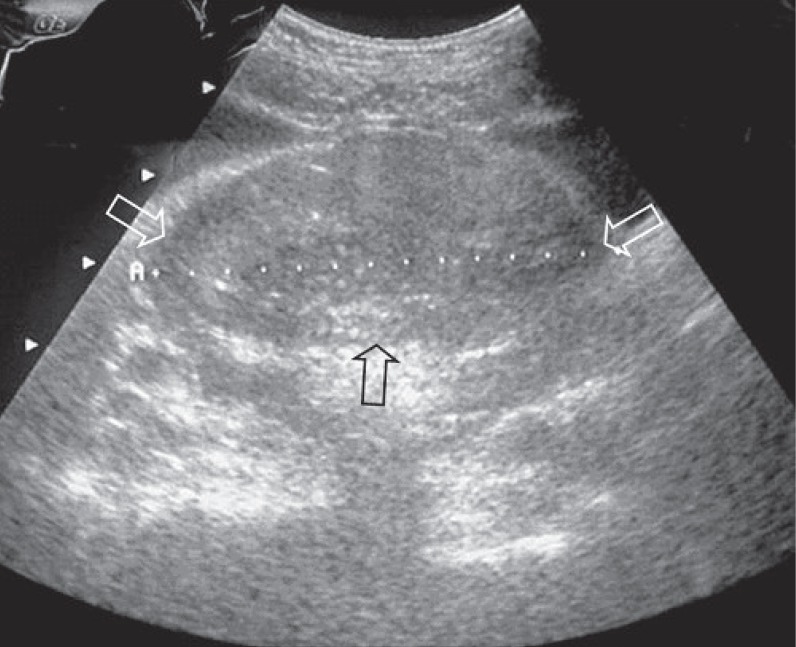
Pelvic ultrasound shows a 8 cm solid,heterogeneous, pelvic mass (arrow) to the posterior side of the urinary bladder.

Ultrasonography of the abdomen showed a voluminous (8 cm in maximum diameter) solid, heterogeneous, pelvic mass to the posterior side of the urinary bladder, probably arising from the small intestine (Fig. 1).

A moderate amount of free fluid in the right iliac fossa was revealed. As a result of the symptoms and radiological findings, preoperative diagnosis of peritonitis due to small intestine perforation and MD tumor with intratumoral necrosis was performed. The patient underwent an emergency surgical resection of the 4 cm ileum with MD neoplasm and lateral ileo-ileal anastomosis.

The surgical specimen showed a well-circumscribed mass, measuring 8 × 3 cm, arising from the MD wall.

**Figure 2:**
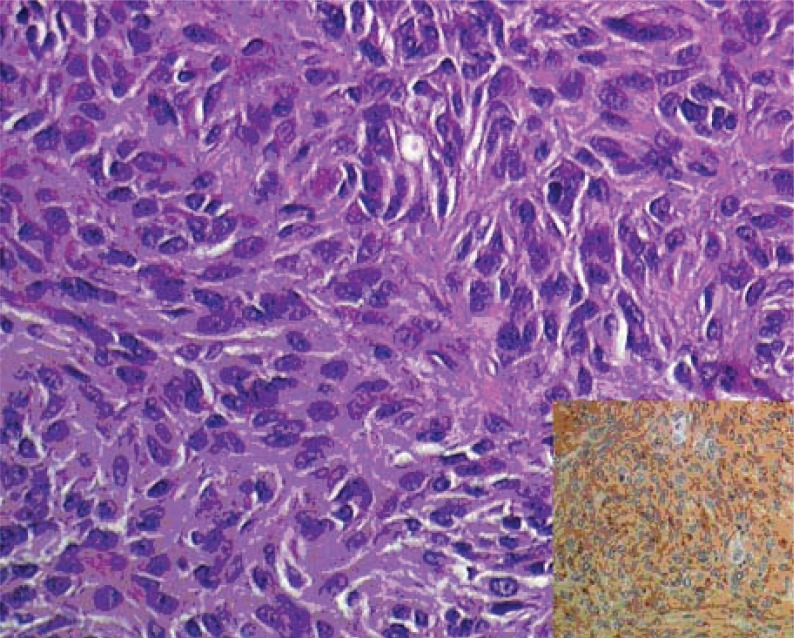
Spindle and epithelioid tumor cell were pleomophic (H&E 200X). Insert: strongly positive reaction for the antibody CD117 (c-kit) was diffusely seen in the neoplasm (200X).

The pathology report was of high risk GIST (mitotic rate >10/50 high power field, necrosis and 8 cm in maximum diameter) (Fig. 2). All margins were negative.

Twelve hours after surgery a fecal peritonitis due to perforation of the perianastomotic ileum was revealed.

A second surgical operation with latero-lateral ileo-ileal anastomosis was performed.

After 10 days the patient was discharged in good condition. Abdominopelvic helical Computerized Tomography (CT), performed 8-months after surgery, revealed a voluminous mass (10.5 cm in maximum diameter) in the right iliac fossa and two contiguous masses of 10 cm and of 2.8 cm in maximum diameter in the pelvis.

**Figure 3:**
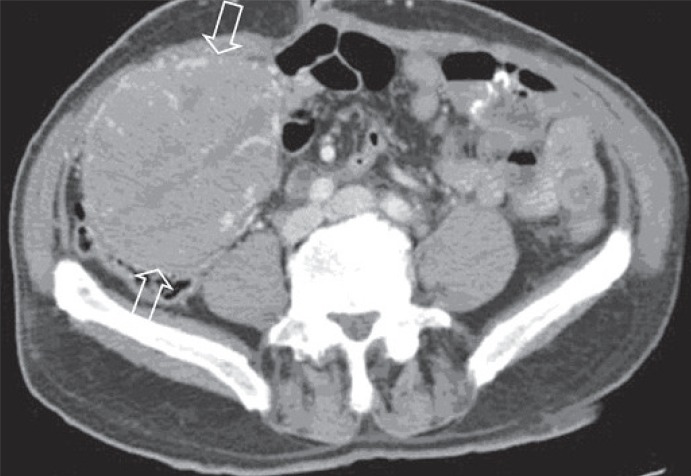
Abdominopelvic helical Computed Tomography, performed 8-months after surgery, revealed a voluminous mass in the right iliac fossa (arrow). After intravenous contrast material administration the masses showed heterogeneous enhancement and well-defined margins.

On precontrast CT the masses were solid with heterogeneous content, well-defined margins and heterogeneous enhancement after intravenous contrast material administration (Fig. 3). No distant or lymph nodes metastases were revealed.

The patient was treated with imatinib mesylate (400 mg/die for 4 weeks) and he did not present adverse effects to the therapy.

**Figure 4:**
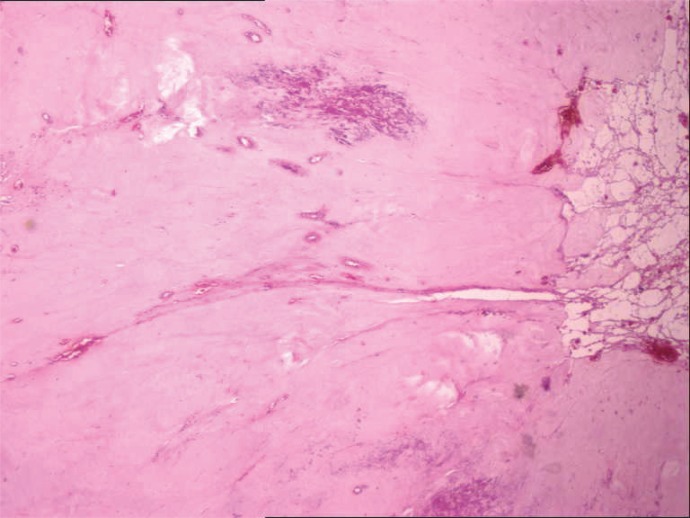
Regressive features as fibrosis and necrosis due to the therapy with the tyrosine kinase inhibitors (H&E 200X).

Abdominopelvic helical CT, performed 18 months after surgery, revealed marked reduction in size of the masses. The histology of these masses showed regressive features as fibrosis and necrosis due to the therapy with the tyrosine kinase inhibitors (Fig. 4).

Appearance and size of the lesions did not change on CT scans obtained 5-years after surgery.

In January 2008 the patient was referred to our institution because of fecal occult blood.

Abdominopelvic helical CT, showed increase in size (11 cm in maximum diameter) of the mass localized in the upper abdomen and multiple hepatic metastases. The abdominal and pelvic masses were resected. The histology showed a proliferative pattern of high risk GIST metastases.

The patient was discharged from the hospital 9 days after surgery.

## DISCUSSION

Today, on the basis of the pathological and immunohistochemical features, most gastrointestinal mesenchymal tumors are classified as GISTs (13).

Since other mesenchymal tumors as leiomiosarcomas (14), fibrosarcomas (15) and not other specified stromal tumors arising from MD have been reported more frequently than GIST, probably the application of the new histological and immunohistochemical techniques could modify the original diagnosis.

In our case the outcome shows that the site is very important in determining the prognosis. Patients with a small bowel localization do worse than those with stomach GIST as reported by DeMatteo *et al* (16).

In a case of a MD localization, the treatment with imatinib mesylate has been reported by Khoury II *et al* (8), but the impact on the clinical behaviour of disease has not been described. In the present report the metastatic progression of disease demonstrated the value of prognostic indicators for assessing risk of aggressive behaviour of GIST also in MD, despite the small number of cases reported. Imatinib mesylate controlled the disease and for the first time we document the histological effects of the therapy.

CT is a valuable procedure for detection of local recurrence, distant metastases and for surveillance after surgery. If a localized recurrence is detected, the patient may be treated with repeated resection to prevent complications and to attempt a cure.

In conclusion, our case illustrated the first long term-follow-up in a high-grade GIST in MD and the histological features of the treatment with imatinib mesylate.
